# Downregulated M6A modification and expression of circRNA_103239 promoted the progression of glioma by regulating the miR-182-5p/MTSS1 signalling pathway

**DOI:** 10.7150/jca.85320

**Published:** 2023-10-16

**Authors:** Shoudan Zhang, Peng Zhang, Anyi Wu, Zhipeng Xu, Shilu Huang, Xinglei Liu, Jun Dong

**Affiliations:** 1 Department of Neurosurgery, The Second Affiliated Hospital of Soochow University, Suzhou 215004, Jiangsu, China.; 2 Department of Neurosurgery, the First Affiliated Hospital of Jinzhou Medical University, Jinzhou 121000, Liaoning Province, P.R. China; 3 Department of Neurosurgery, People's Hospital of Rugao, Jiangsu, China.

**Keywords:** M6A modification, circRNA_103239, glioma, miR-182-5p, MTSS1

## Abstract

Glioma is a common type of tumor in the central nervous system, and the mortality is high. The prognosis of advanced glioma patients remains poor, and the therapeutic strategies need to be developed. The roles of circRNAs in glioma remain largely unknown. The aim of this study was to explore the functions circRNA_103239 in the biological behaviour changes of glioma cells. The expression of circRNA_103239 in clinical samples and glioma cells were examined using RT-qPCR. The targets of circRNA_103239 were predicted using bioinformatics approach. Gain- and loss-of-function study were carried out. The proliferation of transfected cells were evaluated by CCK-8 assay. Migratory and invasive activities of the cells were examined using wound healing, colony formation and transwell assay. Tumor growth was also evaluated in vivo. The results indicated that the expression of circRNA_103239 was predominantly detected in the cytoplasma of glioma cells. In addition, the expression of circRNA_103239 was down-regulated in glioma, and up-regulated circRNA_103239 inhibited the progression of glioma. Furthermore, miR-182-5p was the novel target of circRNA_103239 in glioma, and MTSS1 was the putative downstream molecule of circRNA_103239/miR-182-5p axis. Additionally, circRNA_103239 suppressed the progression of glioma in a miR-182-5p/MTSS1 dependent manner. Moreover, circRNA_103239 inhibited tumour growth in vivo, and the expression of circRNA_103239 was regulated by METTL14-mediated m6A modification. In summary, in normal cells, METTL14 mediated the m6A modification and expression of circRNA_103239, which sponging miR-182-5p and inducing the expression of MTSS1, subsequently inhibiting the EMT; whereas in glioma cells, downregulated METTL14 induced downregulated m6A modification and expression of circRNA_103239, further resulting in the up-regulation of miR-182-5p and down-regulation of MTSS1, consequently promoting the EMT of glioma cells and triggering the progression of tumor.

## Introduction

Glioma is a common type of aggressive malignancy in the central nervous system. Although the therapeutics methods of glioma have been developed by introducing chemo-/radio-therapy and targeted biological therapy, the overall survival rates of glioma patients especially those with advanced glioma are still poor 1,2. Therefore, it is urgent to develop the treatments of glioma.

Due to the lack of poly (a) tail, circRNA is rarely found in previous RNA expression studies, and it is considered to be an inert shear product for a long time due to its long low expression level (3,4). However, in recent years, circRNA has been found to have a high expression level in some cells and tissues with the help of relevant biological information analysis methods. More than 10% of the tested cells and tissues have the ability to express circRNA, which has become a hot research topic 3,4. A growing number of sequencing studies indicate that circRNAs are playing an increasingly important role in the pathogenesis of various types of tumors including glioma 5-9. However, the functions of circRNAs in glioma remain largely unknown.

MicroRNAs (miRNAs) are a group of non-coding RNAs and the novel targets of circRNAs. MiRNAs functions through binding to the 3' untranslated region of mRNAs targets. Dysregulation of miRNAs in tumor such as glioma have been reported. For instance, miR-93 could promote the development of glioma cells via PI3K/AKT signaling 10. In addition, miR-140 and miR-152 are involved in the regulation of biological behaviours of glioma cells 11,12. Recent studies have shown that the interaction between circRNAs and miRNAs was involved in the regulation of disease development 13,14. For instance, downregulation of circPVT1 repressed HCC cell growth by upregulating miR-3666 to inhibit SIRT7; the increase in FAM83A expression caused by circ-ZKSCAN1 overexpression could in turn promote the expression of circ-ZKSCAN1; circ-001680 could promote the cancer stem cell population in CRC and induce irinotecan therapeutic resistance by regulating the miR-340 target gene BMI1 15-17. Therefore, we aimed to explore the functions of circRNAs and its downstream molecules in the pathogenesis of glioma.

N6 methyl adenosine (M^6^A) was an RNA modification method widely found in eukaryotic mRNAs and lncRNAs, and M^6^A modification could regulate the expression of genes 18. The level of RNA m6A modification was regulated by both methyltransferase and demethylase. M6A presented dynamic changes in different tissues and different pathological processes. It participated in the formation and development of various diseases 19-21. For instance, it is involved in the unset and progression of cancer, suggesting its biological and translational implications 19. As part of the pathogenesis of influenza infection, influenza virus mRNA is posttranscriptionally methylated at internal adenosine residues to form M6A 21. More importantly, m6A regulators are abnormally expressed and are extensively involved in the progression of glioma by targeting non-coding RNAs. Moreover, as the most important epigenetic regulators, non-coding RNAs can also affect the function of m6A regulators in glioma 22-24. It has confirmed that m6A can regulate circRNA metabolism, including circRNAs biogenesis, translation, degradation and cellular localization 25. However, M^6^A modification on circRNAs remains largely unknown. Metastasis suppressor protein 1 is a protein that in humans is encoded by the MTSS1 gene. MTSS1 could participate in cancer progression or tumor metastasis in a variety of organ sites, most likely through an interaction with the actin cytoskeleton.

In this study, the regulatory roles and novel downstream mechanisms of circRNA_103239 in the biological behaviour changes of glioma cells were evaluated. The expression of circRNA_103239 in clinical samples and glioma cells were examined using RT-qPCR. The targets of circRNA_103239 were predicted using bioinformatics approach. Gain- and loss-of-function study were carried out. The proliferation of transfected cells were evaluated by CCK-8 assay. Migratory and invasive activities of the cells were examined using wound healing, colony formation and transwell assay. Tumor growth was also evaluated in vivo. The results suggested that in normal cells, METTL14 mediated the m6A modification and expression of circRNA_103239, which sponging miR-182-5p and inducing the expression of MTSS1, subsequently inhibiting the EMT; whereas in glioma cells, downregulated METTL14 induced downregulated m6A modification and expression of circRNA_103239, further resulting in the up-regulation of miR-182-5p and down-regulation of MTSS1, consequently promoting the EMT of glioma cells and triggering the progression of tumor. Therefore, the circRNA_103239/miR-182-5p/MTSS1 axis could be a novel therapeutic target for the treatment of glioma.

## Methods

### Characterization of circRNAs of circRNA_103239 in glioma cells

Random and oligo-dT primers were added into extracted RNA for reverse transcription, the expression levels of linear and circular RNAs were examined. 2 μg of Total RNA was incubated with 3 U/μg of RNase R (Epicentre Technologies, Madison, USA) at 37 °C for 30 mins. Following the treatment with RNase R, the RNA expression levels were examined by RT-qPCR. The subcellular localization of circRNA_103239 was carried out.

### Sub-cellular localization of circRNA_103239

Nuclear and cytosolic fractions were separated using cytoplasmic and nuclear RNA purification kit (cat. no. 21000, Norgen Biotek Corp.), and the expression of GAPDH and circRNA_103239 in the fractions were evaluated using RT-qPCR.

### Patient samples

Fifty glioma patients were enrolled in this study. Glioma samples and paired non-cancerous specimens were received from our Hospital from August 2011 to September 2015. Written informed consents were obtained from the patients. Patients were classified according to the 2021 WHO grading scale. Our study was approved by local ethics committee. The experiments were conducted ethically in accordance with the World Medical Association Declaration of Helsinki.

### RNA fluorescence in situ hybridization (FISH)

The expression and localization of circRNA_103239 and miR-182-5p in glioma cells were evaluated using FISH analysis. Biotin-labeled probe for circRNA_103239 (red fluorescence) and digoxin-labeled probe for miR-182-5p (green florescence) were synthesized. Cell suspension was seeded on glass slides and fixed using 4% paraformaldehyde. After dehydration with 70, 95 and 100% ethanol, hybridization was performed at 37 °C overnight in a dark moist chamber. Following hybridization, slides were washed with 50% formamide three times and counterstained with DAPI. The images were captured using a fluorescence microscopy (Leica, SP8 laser confocal).

### RNA extraction and RT-qPCR

Trizol reagent (Sobao Biotechnology, Shanghai, China) was used to extract total RNA from tissues and cells. Quality of extracted RNA was evaluated by bioanalyzer (2100, Agilent). cDNA was synthesized according to the instructions of primescript RT Kit (Takara, Dalian, China); PCR was performed using iTaqTM universal SYBR ® green Supermix (Bio-rad, Hercules, CA). For miRNA, the extraction was performed using miRNeasy Mini Kit (Qiagen, Duesseldorf, Germany), and then reverse transcription was carried out using miScript II RT Kit (Qiagen), and cDNA amplification was performed by miScript SYBR Green PCR Kit (Qiagen) according to the manufacturer's protocol. The primer pairs were designed by Xinbei Biotechnology (Shanghai, China). The primer sequences were as follows: circRNA_103239 Forward (F) 5'-TCATCTGGACCCTCCTCCTCCCCG-3', Reverse (R) 5'-CTTCCTGGGGGACTTTGTGGTGGG-3'; miR-182-5p (F) 5'-ACACTCCAGCTGGGTTTGGCAATGGTAGAACT-3', (R) 5'-TGGTGTCGTGGAGTCG-3'; MTSS1 (F) 5'-CGGCCAGTGATTGAAGAAGAA-3', (R) 5'-TTCTTCTTCAATCACTGGCCG-3'; Vimentin (F) 5'-TGTCCAAATCGATGTGGATGTTTC-3', (R) 5'-TTGTACCATTCTTCTGCCTCCTG-3'; N-cadherin (F) 5'-GCGTCTGTAGAGGCTTCTGG-3', (R) 5'-GCCACTTGCCACTTTTCCTG-3'; E-cadherin (F) 5'-CGACAAAGGACAGCCTATTT-3', (R) 5'-AGTTGGGAAATGTGAGCAAT-3'; PCNA (F) 5'-GACACATACCGCTGCGATCG -3', (R) 5'- TCACCACAGCATCTCCAATAT -3'; GAPDH (F) 5'-ACCACAGTCCATGCCATCAC-3', (R) 5'-TCCACCACCCTGTTGCTGTA-3'; U6 (F) 5′-CTCGCTTCGGCAGCACA-3′, (R) 5′-AACGCTTCACGAATTTGCGT-3′. The relative expression levels were normalized by GAPDH or U6. The 2^-ΔΔCt^ method was used to evaluate the expression. The program used for qPCR was: 95˚C for 5 min, 45 cycles of 95˚C for 15s, 60˚C for 20s and 72˚C for 10s.

### Cell culture

Normal human astrocytes (NHA) and glioma cell lines SNB19, U87, A172, and U251 were obtained from the American Type Culture Collection (Manassas, VA, USA) and cultured in RPMI-1640 medium supplemented with 10% FBS, 100 µg/ml streptomycin and 100 U/ml penicillin (all purchased from GE Healthcare Life Science). Cells were maintained in a humid incubator at 37˚C supplied with 5% CO_2_.

### Cell transfection

In order to produce the knockdown model of circRNA_103239 and MTSS1, shRNAs against circRNA_103239 (sh-circRNA_103239), MTSS1 (sh-MTSS1) as well as the negative control (sh-NC) were obtained from Genepharm Co. Ltd. (Shanghai, China). Annealed shRNA segment was inserted in pU6-Luc-Puro lentivirus vector (Genepharm Co. Ltd.). To generate the cell model with circRNA_103239 overexpression, wildtype (oe-circ_0001946)/mutant (oe-NC) sequence was amplified by PCR, which was subsequently cloned into PLCDH-cir vector (Invitrogen; Thermo Fisher Scientific, Inc., Waltham, MA, USA). In order to generate miR-182-5p up- and down-regulation cell models, miR-182-5p mimics and inhibitors were obtained from Genepharm Co. Ltd. Up-/down-regulation of corresponding molecules were evaluated by RT-qPCR. Cell transfection was carried out by assistance of Lipofectamine^®^2000 (Invitrogen; Thermo Fisher Scientific, Inc.). After 12 hr, culture media was replenished with DMEM containing 10% FBS.

### CCK-8 assay

Cells with a density of 5x10^4^cells/well were inoculated on 96 wells plate. Then, cell proliferation was examined at Day 1, 2, 3 and 4. In brief, 10μL of CCK-8 reagent (Dojindo Molecular Technologies, Inc., Kumamoto, Japan) was added to the cells. Cells were incubated for another 2 hr, the absorbance (450nm) was measured using a microplate reader (Bio-Rad Laboratories, Inc., Hercules, CA, USA).

### Immunofluorescence staining of PCNA

Cells were fixed using ice-cold acetone (SigmaAldrich, Poole, UK) for 15 mins. Following fixation, cells were rinsed with PBS. Subsequently, cells were incubated with blocking solution for 30 mins and then with primary PCNA antibody (1:200; cat. no. 13110; Cell Signaling Technology) overnight in cold room. Next day, cells were rinsed and incubated with Alexa-Fluor 568 labelled secondary antibody (1:1000, Molecular Probes, Eugene) for one hr in dark. Secondary antibody alone group was used as negative control. Then, cell nucleus was stained using DAPI reagent (Vector Laboratories, Peterborough, U.K.). Subsequently, cells were rinsed and mounted onto glass slide by Mowiol reagent with 10% Mowiol D488 (Calbiochem, Nottingham, U.K.), and slides were stored in freezer. Staining was visualized with a Leica DMLB Microscope. Cell images were acquired using a CCD camera (Cool-SNAP-Pro; Media Cybernetics, USA) with Image-Pro Plus software (version 6.0; Media Cybernetics, USA).

### Wound healing assay

The migration of transfected glioma cells was evaluated by wound healing assay. 1x10^6^ cells were seeded into a 6-well plate and cultured for 24hrs until the cells reached 90% confluence. Then, a straight scratch was generated using a sterile pipette tip. Then the cells were washed using PBS and cultured at 37°C with the supply of 5% CO2 for 24 hours. The migration of cells was monitored and the images were captured at 0- and 24-hour using microscope (Leica DFC300FX).

### Colony formation assay

Transfected glioma cells were seeded into a 6- well plate at the density of 500 cells per well, then the cells were cultured in a humidified incubator at 37°C with the supply of 5% CO2 for two weeks. After culture, the cells were fixed with 4% paraformaldehyde and stained with 0.1% crystal violet for 1 hour. Then, colonies were counted using ImageJ (version 1.47; National Institutes of Health, Bethesda, MD, USA).

### Transwell assay

For cell migration, 3x10^5^ cells were well re-suspended in culture media without FBS and seeded in the upper chamber with pore size of 8µm (BD Biosciences, Franklin Lakes, New Jersey, USA). Subsequently, 500 µl culture media containing 10% FBS was added into the lower chamber. Following two days incubation, non-migratory cells were scratched by cotton swab. Cells in lower chamber were fixed with methanol for 10 min and stained using crystal violet (0.5%). Cells were then counted in five randomly selected fields under an inverted light microscope (magnificationx100, Olympus Corporation, Tokyo, Japan).

### Western blotting

Proteins were extracted with RIPA lysis buffer (Beyotime Institute of Biotechnology, Shanghai, China). Protein concentration was measured using BCA kit (Beyotime Institute of Biotechnology). Same amount of protein samples (~30 μg) were subjected to 12% SDS-PAGE, and samples were transferred to PVDF membrane (EMD Millipore, Billerica, MA, USA). Subsequently, the membranes were blocked in PBS supplemented with 5% skimmed milk for one hr and incubated with primary Vimentin (1:1000; cat. no. 5741; Cell Signaling), N-cadherin (1:1000; cat. no. 13116; Cell Signaling), E-cadherin (1:1000; cat. no. 3195; Cell Signaling), MTSS1 antibody (1:1000; cat. no. 4386; Cell Signaling), or GAPDH antibody (1:1,000; cat. no. sc-32233; Santa Cruz Biotechnology Inc.) at 4˚C overnight. The following day, membranes were incubated with HRP-labelled anti-mouse (1:5,000; cat. no. 7076; Cell Signaling) or anti-rabbit IgG (1:5,000; cat. no. 7074; Cell Signaling) for one hr. Blot was visualized with ECL kit (Pierce Biotechnology; Thermo Fisher Scientific, Inc). Protein bands were analyzed by Image J software (NIH, Bethesda, MD, USA).

### Bioinformatics analysis and dual-luciferase reporter assay

The putative downstream molecules of circRNA_103239 were predicted using bioinformatics approach CircNet, Circular RNA Interactome, miR-base database (http://www.mirbase.org/) and TargetScan-Human7.1 (http://www.targetscan.org/vert_71/). Wild-type (WT-circRNA_103239 or WT-MTSS1) sequence of the 3'-UTR carrying novel binding sites of miR-182-5p were purchased from Saicheng Biotechnology, Guangzhou, China. and were then integrated into pGL3 Vector (Saicheng Biotechnology, Guangzhou, China). QuikChange Multi Site-Directed Mutagenesis assay (Stratagene; Agilent) was carried out to generate MUT-circRNA_103239/MUT-MTSS1 reporter carrying mutant miR-182-5p binding sequence. The plasmids were co-transfected with miR-NC or miR-182-5p mimics into cells. Luciferase activity was examined 48 h after transfection by a Dual Luciferase Reporter gene detection system (Hongli Biotechnology, Shanghai, China). The activity of firefly luciferase was normalized using Renilla luciferase.

### RNA-RNA pull-down assay

Biotin-labelled probe of circRNA_103239 and negative control were obtained from GenePharma (Shanghai, China). Cell lysates were labeled using Dynabeads M-280 Streptavidin. Beads with immobilized circRNA_103239 were then treated with 10mM ethylenediaminetetraacetic acid. Bound RNAs were extracted using TRIzol^®^ reagent and amplified by RT-qPCR.

### RNA immunoprecipitation (RIP) assay

The RIP assay was carried out using RNA-Binding Protein Immunoprecipitation Kit (cat. no. 17-700, Millipore) and HiS- cript II qRT SuperMix II kit (cat. no. R223, VAZYME). Anti-Ago2 (cat. no. ab5072, Cambridge, MA, USA) and anti-m6A antibody (cat. no. A-1801-020, Epigentek) were used for RIP and M^6^A RIP assays. RIPA buffer solution was used to dissolve the cells, then incubated at 4℃ for 4h with primary antibody or anti rabbit IgG, and finally magnetic beads were added at 4℃ overnight. RNA was purified from magnetic bead binding RNA protein complex and analyzed by qPCR.

### In vivo experiment

Male athymic nude mice at the age of six to eight weeks old were used. Glioma cells were transfected with LV-NC or LV-circRNA_103239. Each mouse was subcutaneously inoculated with 1x10^7^ cells resuspended in PBS. After the development of tumor, the tumor volume was monitored every 7 days. Tumor volume was calculated using formula: volume=(length x width^2^)/2. At 42 days after inoculation, the mice were sacrificed. The in vivo experiments were carried out according to the national and international guidelines and the protocol was approved by local ethics committee.

### Statistics analysis

Data were presented as means ± standard error, and data analysis was performed using SPSS software (version 25.0; SPSS, Inc., Chicago, USA). The significance of differences was evaluated by Student's t-test or one-way analysis of variance (ANOVA). The expression of relevant molecules in para-carcinoma and glioma samples were compared using paired t-test. Post-hoc Tukey test was performed after ANOVA. The association within RNA expression was studies by Pearson's correlation analyses. Three repeats have been done for each experiment. P<0.05 indicated a statistically significant difference.

## Results

### Characterization of circRNAs and subcellular localization of circRNA_103239 in glioma cells

**Figure 1:**
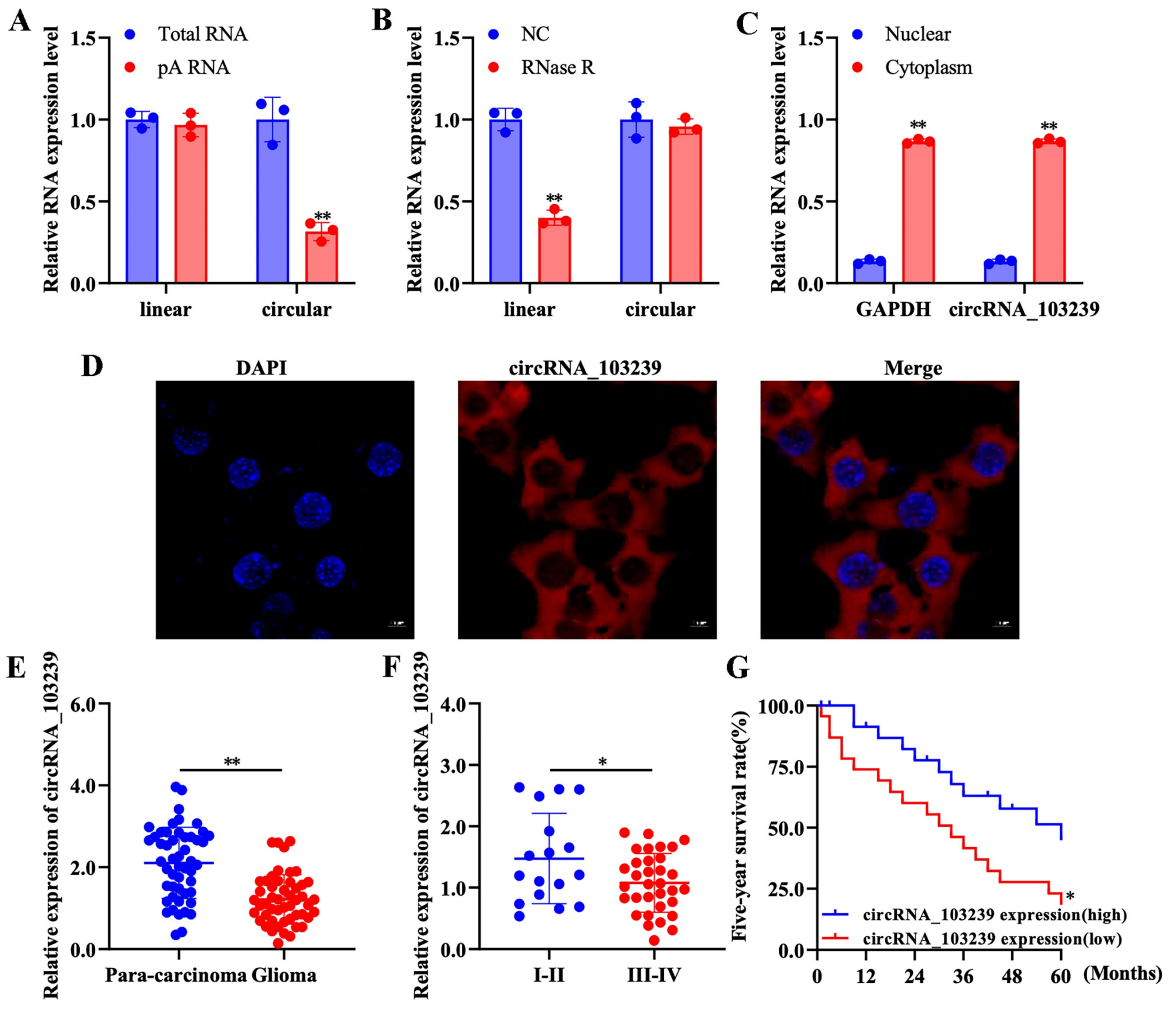
** Characterization of circRNAs and subcellular localization of circRNA_103239 in glioma cells.** A. The expression of linear RNAs was detected after adding oligo-dT primer, while the expression of circRNAs was not detected. B. CircRNAs were resistant to RNase R-induced degradation compared to the linear RNAs. C. CircRNA_1032339 was detected in the cytoplasmic fraction. D. The localization of circRNA_103239 in cytoplasma was also confirmed by FISH analysis. E. The expression of circRNA_103239 was significantly reduced in glioma samples compared with the controls. F. The levels of circRNA_103239 was down-regulated in glioma patients with advanced tumour compared to those at early stage. G. Patients with low circRNA_103239 expression exhibited poor overall survival. Three repeats have been done for each experiment. *p<0.05, **p<0.01 vs. corresponding control.

In order to characterize the circRNAs in glioma cells, function studies were performed. The expression of linear RNAs was detected after adding oligo-dT primer, while the expression of circRNAs was not detected (Fig. 1A). In addition, circRNAs were resistant to RNase R-induced degradation compared to the linear RNAs (Fig. 1B). As indicated in our previous microarray data, circRNA_103239 was selected for further study and its subcellular localization was also investigated; it was detected in the cytoplasmic fraction (Fig. 1C). Furthermore, the localization of circRNA_103239 in cytoplasma was also confirmed by FISH analysis (Fig. 1D).

### The expression of circRNA_103239 was down-regulated in glioma

**Figure 2:**
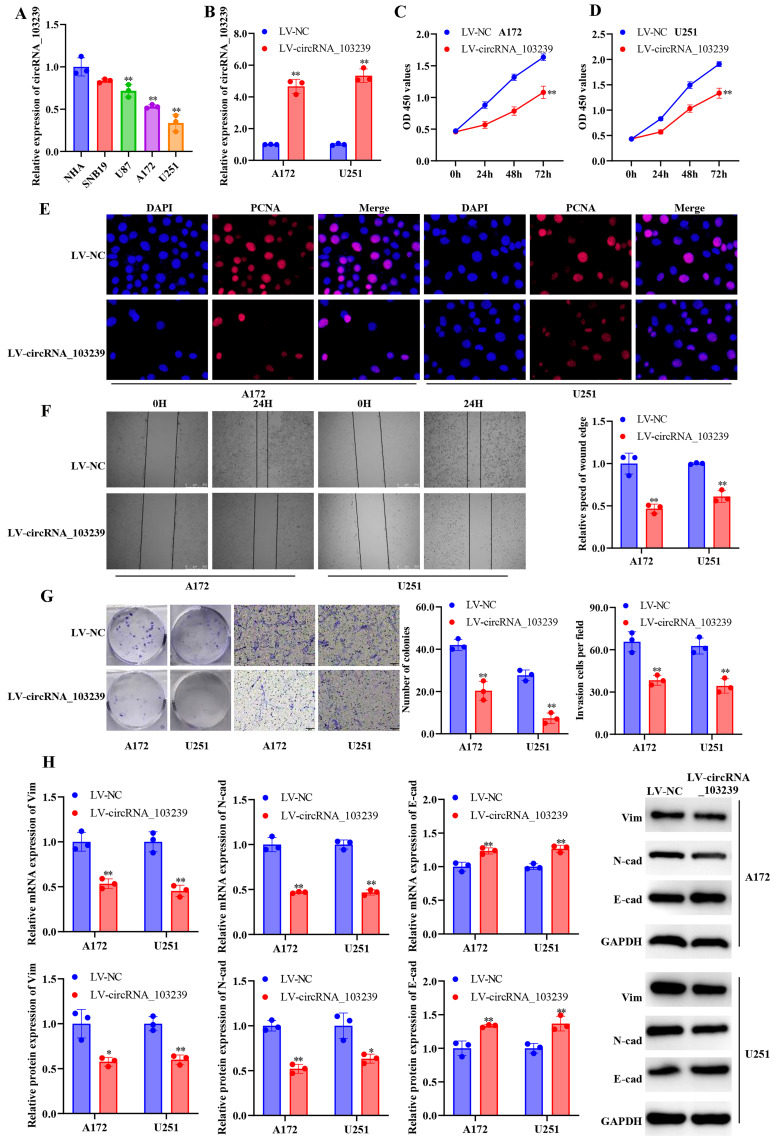
** The expression of circRNA_103239 was down-regulated in glioma, and up-regulated circRNA_103239 suppressed the progression of glioma.** A. The levels of circRNA_103239 were remarkably decreased in glioma cells compared to normal human astrocytes. B. A172 and U251 cells were transfected with LV-circRNA_103239, and the over-expression of circRNA_103239 were confirmed using RT-qPCR. C and D. The proliferation of glioma cells was inhibited after the transfection with LV-circRNA_103239. E. The levels of PCNA were notably down-regulated in cells overexpressing circRNA_103239. F. The migratory ability of glioma cells was significantly reduced after the transfection with LV-circRNA_103239. G. The colony formation ability of glioma cells was remarkably reduced in LV-circRNA_103239 transfected cells; the invasive ability of cells treated with LV-circRNA_103239 was significantly decreased. H. RT-qPCR revealed that the mRNA levels of EMT-associated molecules vimentin and N-cadherin were significantly reduced, while the expression of E-cadherin were remarkably elevated; western blotting suggested that the protein levels of vimentin and N-cadherin were notably decreased, while the expression of E-cadherin were remarkably increased. Three repeats have been done for each experiment. , **p<0.01 vs. NHA or LV-NC.

The levels of circRNA_103239 was examined in glioma specimens and paired para-carcinoma samples. The expression of circRNA_103239 was significantly reduced in glioma samples compared with the controls (Fig. 1E). In addition, the levels of circRNA_103239 was down-regulated in glioma patients with advanced tumour compared to those at early stage (Fig. 1F). Moreover, patients with low circRNA_103239 expression exhibited poor overall survival (Fig. 1G). Furthermore, the levels of circRNA_103239 were remarkably decreased in glioma cells compared to normal human astrocytes (Fig. 2A).

### Up-regulated circRNA_103239 suppressed the progression of glioma

In order to explore the roles of circRNA_103239 on the biological behaviours in glioma cells, A172 and U251 cells were transfected with LV-circRNA_103239, and the over-expression of circRNA_103239 were confirmed using RT-qPCR (Fig. 2B). In the function experiments, the proliferation of glioma cells was inhibited after the transfection with LV-circRNA_103239 (Fig. 2C and D). In consistence with these findings, the levels of PCNA were notably down-regulated in cells overexpressing circRNA_103239 (Fig. 2E). Additionally, the migratory ability of glioma cells was significantly reduced after the transfection with LV-circRNA_103239 (Fig. 2F). Furthermore, the colony formation and invasion ability of glioma cells were remarkably reduced in LV-circRNA_103239 transfected cells (Fig. 2G). Moreover, the expression of EMT-associated molecules vimentin and N-cadherin were significantly reduced, while the levels of E-cadherin were remarkably elevated (Fig. 2H).

### MiR-182-5p was the putative target of circRNA_103239 in glioma

**Figure 3:**
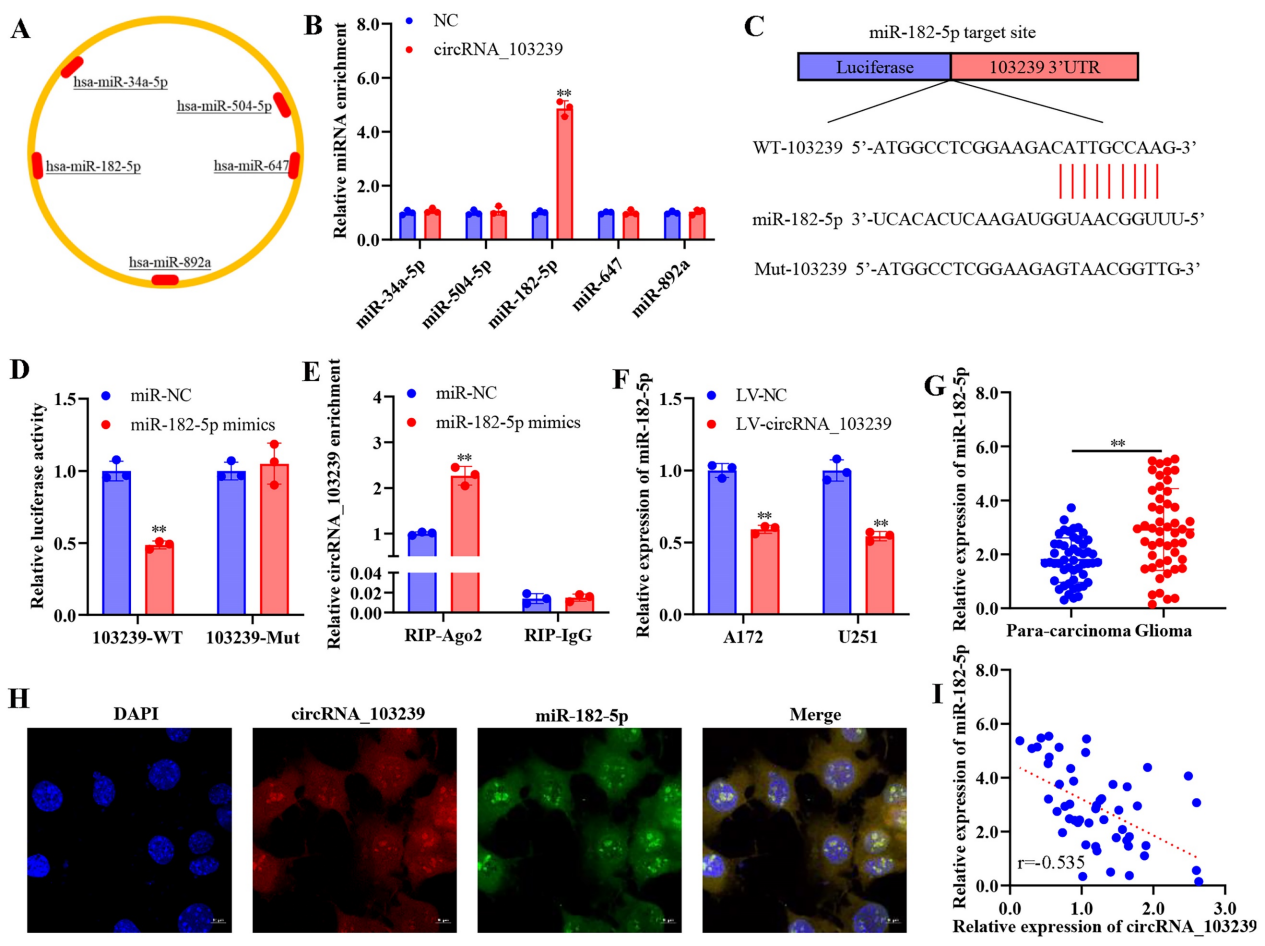
** MiR-182-5p was the putative target of circRNA_103239 in glioma.** A. The downstream molecules of circRNA_103239 was predicted using bioinformatics approach. B. The interaction of circRNA_103239 and miR-182-5p was confirmed using RNA-RNA pull-down assay. C. The potential binding sites between circRNA_103239 and miR-182-5p were predicted. D. The interaction of these tow molecules was confirmed using dual-luciferase reporter assay. E. The binding of circRNA_103239 and miR-182-5p was also validated using RIP assay. F. The expression of miR-182-5p was remarkably reduced in glioma cells transfected with LV-circRNA_103239. G. The levels of miR-182-5p were notably up-regulated in glioma specimens. H. FISH analysis indicated the co-localization of circRNA_103239 and miR-182-5p in U251 cells. I. The expression of circRNA_103239 and miR-182-5p in glioma samples were negatively correlated. Three repeats have been done for each experiment. **p<0.01 vs. miR-NC or LV-NC.

**Figure 4:**
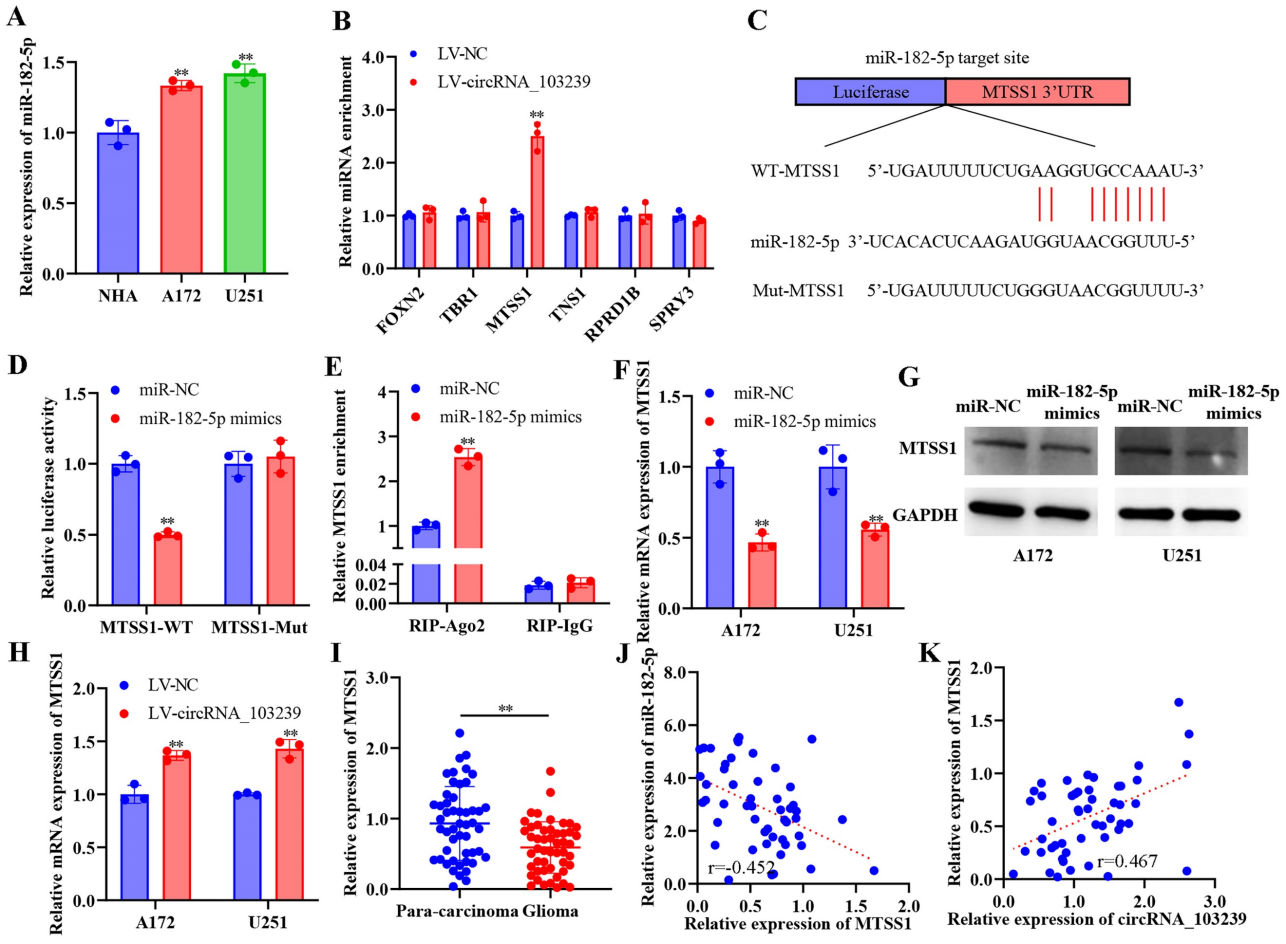
** MTSS1 was the potential downstream molecule of circRNA_103239/miR-182-5p signalling in glioma.** A. The expression of miR-182-5p was significantly up-regulated in glioma cells. B. Among the potential targets, when circRNA_103239 was overexpressed, only the relative miRNA enrichment of MTSS1 was upregulated. C. The potential binding sites between miR-182-5p and MTSS1 was predicted. D. The interaction between these two molecules was confirmed by dual-luciferase reporter assay. E. The binding of miR-182-5p and MTSS1was validated by RIP assay. F and G. The expression of MTSS1 was remarkably reduced at both mRNA and protein levels in glioma cells transfected with miR-182-5p mimics. H. The levels of MTSS1 were notably elevated in glioma cells transfected with LV-circRNA_103239. I. The expression of MTSS1 was significantly decreased in glioma samples compared with para-carcinoma controls. J. The expression of miR-182-5p and MTSS1 was reversely correlated in glioma specimens. K. The expression of circRNA_103239 and MTSS1 was positively correlated in glioma samples. Three repeats have been done for each experiment. **p<0.01 vs. corresponding control.

The downstream molecules of circRNA_103239 was predicted using bioinformatics approach (Fig. 3A). Among the potential targets, the interaction of circRNA_103239 and miR-182-5p was confirmed using RNA-RNA pull-down assay (Fig. 3B). The potential binding sites between circRNA_103239 and miR-182-5p were predicted (Fig. 3C), and the interaction of these tow molecules was confirmed using dual-luciferase reporter assay (Fig. 3D). The binding of circRNA_103239 and miR-182-5p was also validated using RIP assay (Fig. 3E). Moreover, the expression of miR-182-5p was remarkably reduced in glioma cells transfected with LV-circRNA_103239 (Fig. 3F). Additionally, the levels of miR-182-5p were notably up-regulated in glioma specimens (Fig. 3G). Furthermore, FISH analysis indicated the co-localization of circRNA_103239 and miR-182-5p in U251 cells (Fig. 3H). Furthermore, the expression of circRNA_103239 and miR-182-5p in glioma samples were negatively correlated (Fig. 3I). In addition, the expression of miR-182-5p was significantly up-regulated in glioma cells (Fig. 4A).

### MTSS1 was the potential downstream molecule of circRNA_103239/miR-182-5p signalling in glioma

The putative targets of miR-182-5p were predicted using bioinformatics approach. Among the potential targets, when circRNA_103239 was overexpressed, only the relative miRNA enrichment of MTSS1 was upregulated (Fig. 4B). The potential binding sites between miR-182-5p and MTSS1 was predicted (Fig. 4C), and the interaction between these two molecules was confirmed by dual-luciferase reporter assay (Fig. 4D). The binding of miR-182-5p and MTSS1was validated by RIP assay (Fig. 4E).

Moreover, the expression of MTSS1 was remarkably reduced at both mRNA and protein levels in glioma cells transfected with miR-182-5p mimics (Fig. 4F and G). In addition, the levels of MTSS1 were notably elevated in glioma cells transfected with LV-circRNA_103239 (Fig. 4H). Furthermore, the expression of MTSS1 was significantly decreased in glioma samples compared with para-carcinoma controls (Fig. 4I). In addition, the expression of miR-182-5p and MTSS1 was reversely correlated in glioma specimens (Fig. 4J), where the expression of circRNA_103239 and MTSS1 was positively correlated (Fig. 4K).

### circRNA_103239 suppressed the progression of glioma in a miR-182-5p/MTSS1 dependent manner

**Figure 5:**
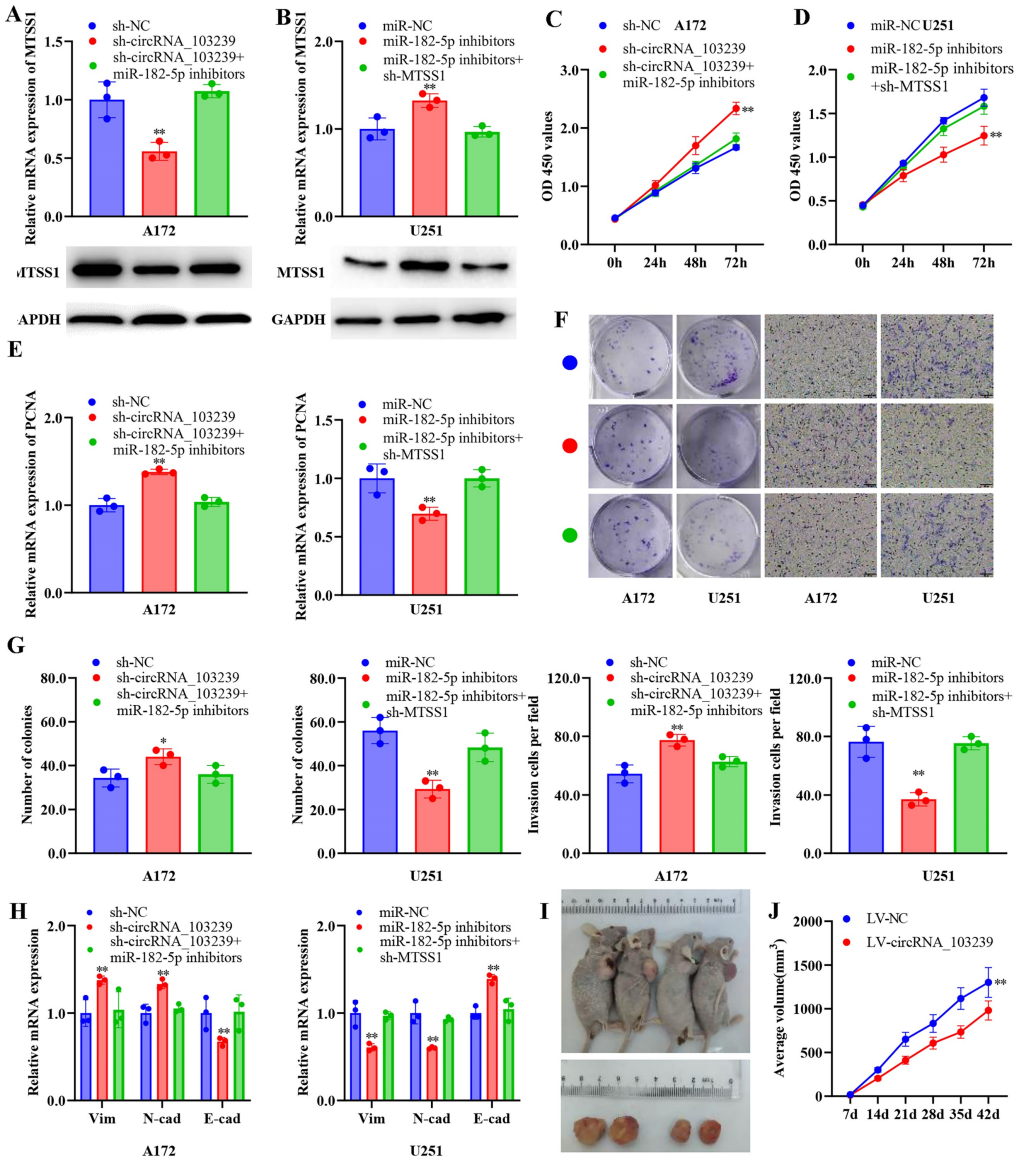
** CircRNA_103239 suppressed the progression of glioma in a miR-182-5p/MTSS1 dependent manner.** A. The expression of MTSS1 was reduced at both mRNA and protein levels in cells transfected with sh-circRNA_103239, which was reversed by the co-transfection with miR-182-5p inhibitors. B. When the cells were co-transfected with sh-MTSS1, the levels of MTSS1 were notably reduced. C. The proliferation of glioma cells was enhanced after the transfection with sh-circRNA_103239, which was reversed in cells co-transfected with miR-182-5p inhibitors. D. Cell proliferation was inhibited after the transfection with miR-182-5p inhibitors, and the effects were abolished following the co-transfection with sh-MTSS1. E. The expression of PCNA exhibited the same trend as the CCK-8 assay. F and G. The colony formation and invasion ability were significantly promoted in glioma cells after the transfection with sh-circRNA_103239, and these effects were reversed in cells co-transfected with miR-182-5p inhibitors; the colony formation and invasion ability were remarkably suppressed in glioma cells transfected with miR-182-5p inhibitors, which was restored by the transfection with sh-MTSS1. H. The levels of vimentin and N-cadherin were remarkably increased in glioma cells transfected with sh-circRNA_103239, where the expression of E-cadherin was down-regulated, after the co-transfection with miR-182-5p inhibitors, the abovementioned effects were reversed; the expression of vimentin and N-cadherin were remarkably reduced in glioma cells transfected with miR-182-5p inhibitors, while the levels of E-cadherin were elevated. After the co-transfection with sh-MTSS1, these effects were all reversed. I and J. Tumour growth was significantly suppressed in LV-circRNA_103239 transfected model compared to the LV-NC control in vivo. Three repeats have been done for each experiment. **p<0.01 vs. sh-NC or miR-NC or LV-NC.

In order to identify the roles of circRNA_103239 and its downstream molecules on the biological behaviours of glioma cells, glioma cells were transfected with sh-circRNA_103239 or co-transfected with miR-182-5p inhibitors. The expression of MTSS1 was reduced at both mRNA and protein levels in cells transfected with sh-circRNA_103239, which was reversed by the co-transfection with miR-182-5p inhibitors (Fig. 5A). In addition, glioma cells were transfected with miR-182-5p inhibitors, where the levels of MTSS1 were remarkably elevated. When the cells were co-transfected with sh-MTSS1, the levels of MTSS1 were notably reduced Fig. 5B). In addition, the proliferation of glioma cells was enhanced after the transfection with sh-circRNA_103239, which was reversed in cells co-transfected with miR-182-5p inhibitors (Fig. 5C). In addition, cell proliferation was inhibited after the transfection with miR-182-5p inhibitors, and the effects were abolished following the co-transfection with sh-MTSS1 (Fig. 5D). In consistence with these findings, the expression of PCNA exhibited the same trend as the CCK-8 assay (Fig. 5E). Furthermore, the colony formation and invasion ability were significantly promoted in glioma cells after the transfection with sh-circRNA_103239, and these effects were reversed in cells co-transfected with miR-182-5p inhibitors (Fig. 5F and G). Moreover, the colony formation and invasion ability were remarkably suppressed in glioma cells transfected with miR-182-5p inhibitors, which was restored by the transfection with sh-MTSS1 (Fig. 5F and G). The levels of vimentin and N-cadherin were remarkably increased in glioma cells transfected with sh-circRNA_103239, where the expression of E-cadherin was down-regulated (Fig. 5H). After the co-transfection with miR-182-5p inhibitors, the abovementioned effects were reversed (Fig. 5H). Similarly, the expression of vimentin and N-cadherin were remarkably reduced in glioma cells transfected with miR-182-5p inhibitors, while the levels of E-cadherin were elevated. After the co-transfection with sh-MTSS1, these effects were all reversed (Fig. 5H).

### CircRNA_103239 inhibited tumour growth in vivo, and the expression of circRNA_103239 was regulated by METTL14-mediated m6A modification

**Figure 6:**
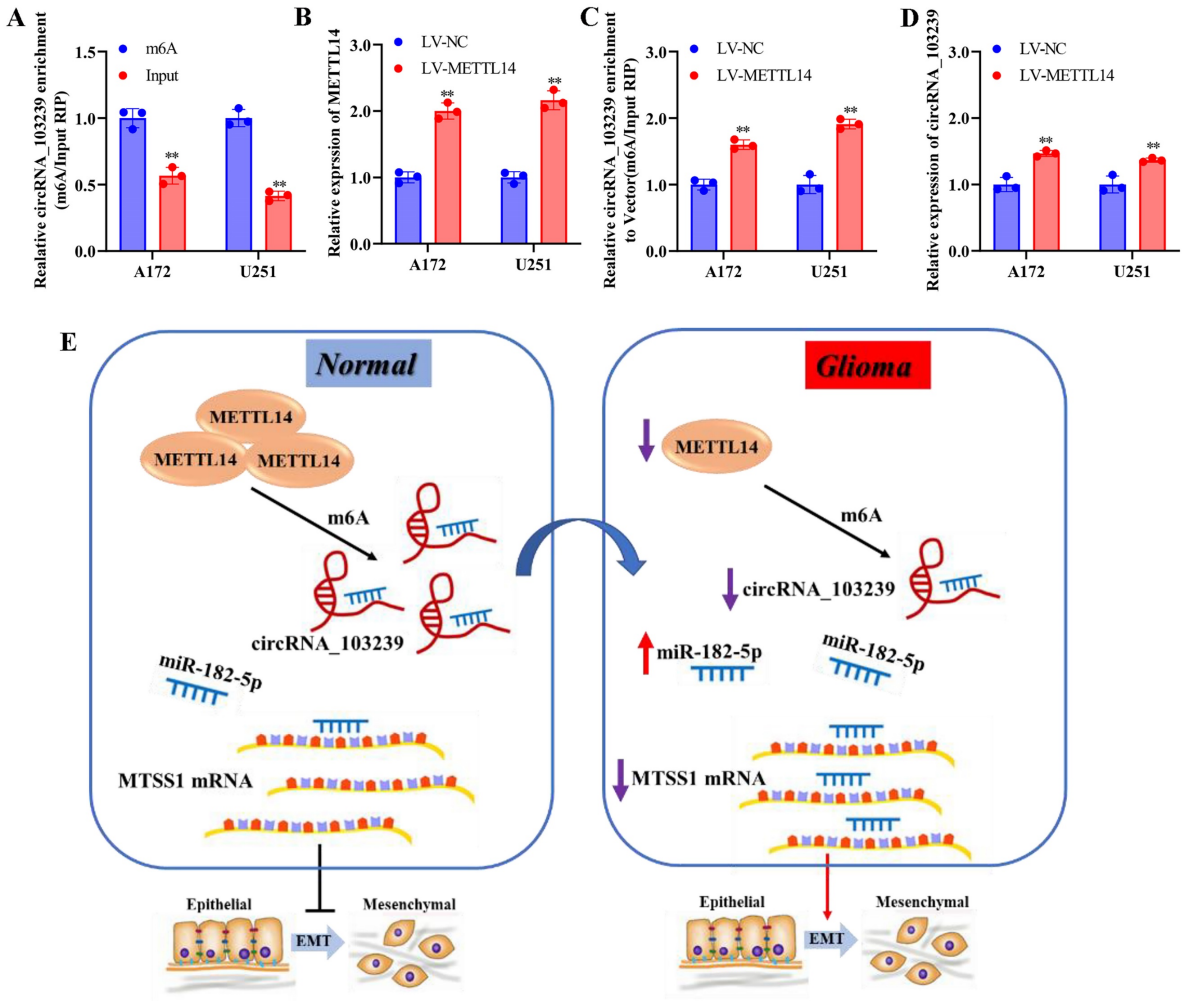
** The expression of circRNA_103239 was regulated by METTL14-mediated m6A modification.** A. RIP assay validated the m6A modification of circRNA_103239 in glioma cells. B-D. the m6A modification and expression of circRNA_103239 were restored in glioma cells transfected with LV-METTL14. E. in normal cells, METTL14 mediated the m6A modification and expression of circRNA_103239, which sponging miR-182-5p and inducing the expression of MTSS1, subsequently inhibiting the EMT; whereas in glioma cells, downregulated METTL14 induced downregulated m6A modification and expression of circRNA_103239, further resulting the up-regulation of miR-182-5p and down-regulation of MTSSA, consequently promoting the EMT of glioma cells and triggering the progression of tumor. Three repeats have been done for each experiment. **p<0.01 vs. m6A or LV-NC.

In the in vivo experiments, tumour growth was significantly suppressed in LV-circRNA_103239 transfected model compared to the LV-NC control (Fig. 5I and J). Furthermore, to investigate the cause of downregulated circRNA_103239 in glioma cells, further function study was carried out. RIP assay validated the m6A modification of circRNA_103239 in glioma cells (Fig. 6A). Furthermore, the expression of METLL14 was increased in glioma cells transfection with LV-METTL14 (Fig. 6B). In addition, the m6A modification of circRNA_103239 were restored in glioma cells transfected with LV-METTL14 (Fig. 6C), and the expression of circRNA_103239 was elevated in the cells after the treatment with LV-METTL14 (Fig. 6D).

## Discussion

In this study, the regulatory roles and downstream targets of circRNA_103239 in the pathogenesis of glioma were explored. The results indicated that the expression of circRNA_103239 was detected in the cytoplasma of glioma cells. In addition, the expression of circRNA_103239 was down-regulated in glioma, and up-regulated circRNA_103239 inhibited the proliferation, migration, invasion and EMT of glioma. Moreover, circRNA_103239 inhibited tumour growth in vivo. Therefore, circRNA_103239 could be a putative tumor suppressor in the development of glioma.

Furthermore, experiments were performed to identify the targets of circRNA_103239 in glioma cells. The results indicated that miR-182-5p was the novel target of circRNA_103239 in glioma, and MTSS1 was the putative downstream molecule of circRNA_103239/miR-182-5p axis. Additionally, circRNA_103239 suppressed the progression of glioma in a miR-182-5p/MTSS1 dependent manner. In consistence with our findings, miR-182-5p was reported as a novel oncogenic factor in numerous types of tumors 26-31. Dysregulation of MTSS1 have been reported in numerous types of cancer, and down-regulation of MTSS1 is correlated poor survival rate 30,31. In consistence with these findings, MTSS1 was also identified as a putative tumor suppressor in glioma cells.

Moreover, the expression of circRNA_103239 was regulated by METTL14-mediated m6A modification. METTL14 is a methyltransferase and the central component of the m6A methylated transferase complex, and it is involved in the dynamic reversible process of m6A modification. Disruption of METTL14-mediated M6A modification has been reported in various types of cancer 32-35. METTL14 could function as tumor suppressor of oncogenic factor during tumour progression. N6 methyl adenosine (M^6^A) was an RNA modification method widely found in eukaryotic mRNAs and lncRNAs, and M^6^A modification could regulate the expression of genes 18. The level of RNA m6A modification was regulated by both methyltransferase and demethylase. M6A presented dynamic changes in different tissues and different pathological processes. It participated in the formation and development of various diseases 19-21. As presented in the Fig. 6E, in normal cells, METTL14 mediated the m6A modification and expression of circRNA_103239, which sponging miR-182-5p and inducing the expression of MTSS1, subsequently inhibiting the EMT; whereas in glioma cells, downregulated METTL14 induced downregulated m6A modification and expression of circRNA_103239, further resulting in the up-regulation of miR-182-5p and down-regulation of MTSS1, consequently promoting the EMT of glioma cells and triggering the progression of tumor.

Accumulating researches have indicated a close association between m6A modification and non-coding RNAs, and suggested m6A-modified non-coding RNAs played a crucial role in tumor progression 36,37. The correlation between m6A modification and non-coding RNAs offers a novel perspective for investigating the potential mechanisms of cancer pathological processes, which suggests that both m6A modification and non-coding RNAs are critical prognostic markers and therapeutic targets in numerous malignancies. M6A modification can influence non-coding RNA metabolism and small molecule inhibitors of m6A-related proteins have great therapeutic potential in human cancer. The putative circRNA_103239/miR-152-5p/MTSS1 axis could be the novel candidate for the targeted therapies in the treatment of glioma.
